# Can universal insecticide-treated net campaigns achieve equity in coverage and use? the case of northern Nigeria

**DOI:** 10.1186/1475-2875-11-32

**Published:** 2012-02-01

**Authors:** Yazoume Ye, Elizabeth Patton, Albert Kilian, Samantha Dovey, Erin Eckert

**Affiliations:** 1 ICF International, 11785 Beltsville Drive, Suite 300, Calverton, MD 20705, USA; 2 Malaria Consortium, Development House, 56-64 Leonard Street, London EC2A 4LT, UK; 3 The US 's President Malaria Initiative/USAID, Washington, USA

## Abstract

**Background:**

Insecticide-treated nets (ITNs) are effective tools for malaria prevention and can significantly reduce severe disease and mortality due to malaria, especially among children under five in endemic areas. However, ITN coverage and use remain low and inequitable among different socio-economic groups in sub-Saharan Africa, particularly in Nigeria. Several strategies have been proposed to increase coverage and use and reduce inequity in Nigeria, including free distribution campaigns recently conducted by the Nigerian federal government. Using data from the first post-campaign survey, the authors investigated the effect of the mass free distribution campaigns in achieving equity in household ownership and use of ITNs.

**Methods:**

A post-campaign survey was undertaken in November 2009 in northern Nigeria to assess the effect of the campaigns in addressing equity across different socio-economic groups. The survey included 987 households randomly selected from 60 clusters in Kano state. Using logistic regression and the Lorenz concentration curve and index, the authors assessed equity in ITN coverage and use.

**Results:**

ITN ownership coverage increased from 10% before the campaigns to 70%-a more than fivefold increase. The campaigns reduced the ownership coverage gap by 75%, effectively reaching parity among wealth quintiles (Concentration index 0.02, 95% CI (-0.02 ; 0.05) versus 0.21 95%CI (0.08 ; 0.34) before the campaigns). ITN use (individuals reporting having slept under an ITN the night before the survey visit) among individuals from households owning at least one ITN, was 53.1% with no statistically significant difference between the lowest, second, third and fourth wealth quintiles and the highest wealth quintile (lowest: odds ratio (OR) 0.87, 95% confidence interval (CI) (0.67 ; 1.13); second: OR 0.85, 95% CI (0.66 ; 1.24); third: OR 1.10 95% CI (0.86 ; 1.4) and fourth OR 0.91 95% CI (0.72 ; 1.15).

**Conclusion:**

The campaign had a significant impact by increasing ITN coverage and reducing inequity in ownership and use. Free ITN distribution campaigns should be sustained to increase equitable coverage. These campaigns should be supplemented with other ITN distribution strategies to cover newborns and replace aging nets.

## Background

Insecticide-treated nets (ITNs) are effective tools for malaria prevention and can significantly reduce severe disease and mortality due to malaria, especially among children under five in endemic areas [1]. However, ITN ownership and use remain low and inequitable among different socio-economic groups in sub-Saharan Africa [2]. With the significant increase in funding in the recent decade, many countries across sub-Saharan Africa are rapidly increasing ITN ownership coverage, that is the percentage of households which own at least one ITN, through several strategies including, social marketing [3,4], free distribution to target groups (through antenatal care (ANC) or immunization campaigns) [5-9], and more recently, free universal population-based distribution campaigns targeting the entire population at risk [3,5,10].

Free distribution campaigns aim at the Roll back Malaria (RBM) goal of universal coverage as opposed to the previous strategies that focused on targeting only pregnant women and children under five. The aim is to achieve the newly agreed-upon Roll Back Malaria (RBM) Partnership target of one ITN for every two people by 2015 [RBM 2008]. Mass ITN distribution campaigns targeting all persons at risk for malaria, particularly in high transmission settings, have the advantage of rapidly achieving high community-level coverage which benefits everyone in the community and not just those who own and sleep under nets [10]. This strategy also has the potential to achieve equity in mosquito net ownership and use, shown by a number of studies in different settings [3,5]; however, the level of achievement depends largely on context specific settings and the effectiveness of the distribution strategy. It is, therefore, important to assess equity in mosquito net ownership and use after each mass distribution in a new setting.

Nigeria is currently engaging in free mass distributions of long-lasting insecticidal nets (LLINs), a type of ITN that is factory-treated and designed to maintain efficacy against mosquito vectors for at least 3 years. The national malaria control strategy plan calls for the distribution of 63 million new LLINs by the end of 2010 and for at least 80% of these nets to be put into use [11].

The first state to implement this new approach was Kano, where several partners, including the U.S. Agency for International Development (USAID), the World Bank, Malaria Consortium, and Support to Nigeria Malaria Programme (SuNMaP), joined forces for implementation. This campaign, which was conducted in two waves in May and July 2009, distributed more than 4 million mosquito nets in State (Malaria Consortium). In Wave 1 of the campaign, which was conducted in May 2009, households with less than nine members received one voucher for two LLINs and those with more members received two vouchers. As a mainly Muslim state, polygamy is common in Kano; therefore, modifications were made in Wave 2 (July 2009) so that each wife and her direct dependents received one voucher for two LLINs.

The urgent need for data to inform policy regarding the scaling up of the campaign strategy drove the decision to carry out a post-campaign evaluation of the first two campaigns implemented in Kano. Using data from the post-campaign survey [12] the authors investigated the effect of the free mass distribution campaign in achieving equity in mosquito net ownership and use.

## Methods

### Study area

**Figure 1:**
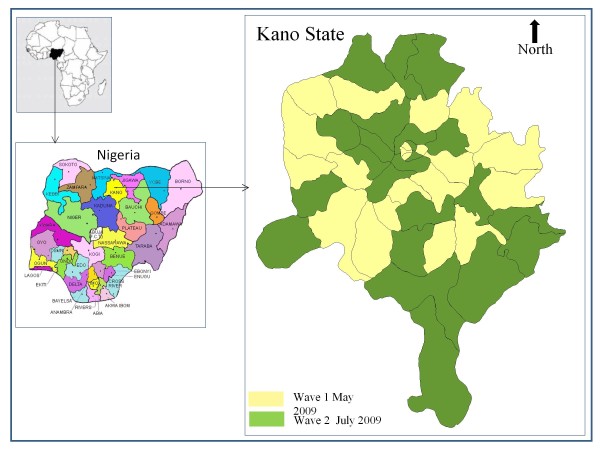
**Location of the study site**.

The study took place in Kano state (Northwest region) and included the 24 local government areas (LGA) covered by the two campaign waves, which took place in May and July 2009, respectively (Figure 1). During Wave 1, the campaign covered 21 LGAs, while during Wave 2, 23 LGAs were covered.

### Study population

This cross-sectional household survey utilized a stratified two-stage cluster sampling design. The strata were the two areas covered in the different campaign waves. Each stratum was considered a survey domain. A total of 60 clusters were selected, including 30 from each stratum. No urban/rural stratification was done but clusters were defined as urban or rural based on their categorization in the 2006 census. Seventeen (17) households were selected from each cluster, resulting in a total sample of 1,020 households in the campaign area.

The sampling procedure of the required number of households was done in two stages including:

#### Stage one: selection of clusters

The household registration lists from the distribution campaigns were used for the selection of clusters. A cluster was defined as a community and selection was carried out independently for each distribution wave using a two-step procedure: first a cumulative list of registered households by ward was compiled and 30 clusters from each of the two strata were selected using systematic sampling with probability proportionate to size (PPS). Second, a list of all communities and the number of registered households was compiled for each selected ward and the required number of villages was selected again using PPS.

#### Stage two: selection of households

Within each selected community 17 households were selected using the following methodology: For small communities (less than 100 compounds), the field team mapped the entire village and from the compiled list of eligible households the supervisor randomly selected 17 households with equal probability for each household. Following the household definition used in the distribution campaign, which was "a wife with her direct dependents", a compound was divided into several households depending on the number of wives. The husband was assigned to the first wife's household. For large communities (more than 120 compounds), the equal size section-approach was used. With the help of local chiefs, the community was divided into sections with approximately equal number of compounds. The supervisor then selected randomly one of these sections within which all households were mapped and selected as above.

### Data collection

Data collection took place from October 19 to November 4, 2009, corresponding to five months after the first campaign and three after the second campaign. The data collection was done using a questionnaire adapted from Malaria Indicator Survey (MIS) Household Questionnaire [13]. The questionnaire was composed of six sections including household roster, household characteristics, campaign net distribution, nets received during the campaign, nets owned by the household, and nets previously owned by the household. The questionnaire was pre-tested in 30 households in a community that was not selected for the survey and corrections were made before the training of the field team.

Prior to the fieldwork, community mobilization activities took place. This was critical; specifically, it attempted to ensure that the researchers did not create further expectation of another distribution campaign after the survey. Such expectations could have potentially influenced some households to under report their mosquito net possession in hopes of receiving additional nets.

Each selected household was visited and the head of household or one of his/her adult dependents was interviewed. In case no appropriate respondent was found at the house, a new visit was scheduled later that day. At least three attempts were made to reach a respondent before dropping the household without replacing it. The main respondent was the head of household or his/her adult dependents except for the section on the mosquito net receipt at the delivery point, for which the person who collected the mosquito net was interviewed.

To ensure high data quality, the team supervisor reviewed all questionnaires daily for completeness and possible inconsistencies and ensured that missing information was corrected while still in the field. In addition, spot-checks were performed on 12% of interviews conducted by each fieldworker.

### Data processing and analysis

Data entry was done using QPS software with double entry of all records. Both data sets were then compared and any discrepant records were verified using the original questionnaires. After the first stage of cleaning, the data set was transferred to STATA 10 for further consistency checks, preparation of data files and analysis.

Two types of analysis were performed. The first one included two binary response logistic regression models. Model 1 assessed the effect of socio-economic status (wealth quintile- highest quintile used as reference) of the household on ITN (including both conventional ITNs and LLINs) ownership after the campaign, controlling for several covariates including campaign wave, education level of head of household, size of household, presence of pregnant women in the household, presence of under fives in the household, and whether the household was present at the mosquito net distribution point. The second model was used to assess the effect of socio-economic status on ITN use of individuals from households which owned at least one ITN. Only members who slept in the household the night preceding the survey visit were included in the analysis. Covariates controlled for included place of residence, gender, education of the head of the household, size of the household, ratio ITN/household member, age, and use of mosquito repellents (aerosol, coils and herbs)

The second set of analyses included equity analysis using the Lorenz concentration curve and index [14] to assess the relative fairness of the distribution in terms of household ITNs ownership and use considering the wealth quintile. The concentration curve plots the cumulative proportion of the outcome variable (ITNs ownership and ITNs use) against the cumulative proportion of the sample population ranked by socio-economic status (wealth quintiles). The curves are compared to the diagonal or equity line. A curve above the diagonal line will indicate the concentration of the outcome among the poor while a curve below will means that the outcome is concentrated among the rich. If there is an equal concentration among poor and rich the curve matches with the diagonal line [14].

The concentration index ranges between -1 and 1. An index of 0 reflects equitable distribution of the outcome between poor and rich while a concentration index of more than 0 suggests that the outcome is more prevalent among the poor. Conversely, a negative index indicates that the outcome is more concentrated among the rich. An index closer to 0 therefore expresses more equitable distribution of the outcome among household with different socio-economic levels [14].

### Ethical clearance

This paper used data from the Kano post mosquito net free distribution campaign survey conducted on the behalf the Federal and State Malaria Control Program. Because this was part of the programmatic activity, ethical clearance was exempted. Informed consent was obtained from each participant.

## Results

### Study population characteristics

Out of 1,020 households sampled, 987 (97.0%) were interviewed. Thirty-three households were not interviewed due to non-availability of an appropriate respondent at home after three visits (31) and refusals (2). For both campaign waves, the coverage of the target sample was equally high (96.5% for Wave 1 and 97.0% for Wave 2). The sample covered a total population of 4,638 of which 50.7% were female and 49.3% were male. The average household size was 4.7 people and the majority (95%) of these households was headed by men. Pregnant women were recorded in 13% of the households and children under the age of five in 62% of the households. Of the 4,638 individuals covered in the samples, 4,602 (99.2%) slept in the household the night preceding the survey visit (de facto population). Of the de facto population, 3,056 (66.4%) individuals belonged to households which owned at least one ITN.

### Household socio-economic level and mosquito net ownership

Table 1 presents the results of the logistic regression assessing the effect of socio-economic status on ITN ownership. The results show that there are no statistically significant differences in household ITN ownership between the highest wealth quintile and the other quintiles. The fourth quintile had the highest odds of owning an ITN, but the difference with the reference did not reach statistical significance (OR 1.48, 95% CI 0.82 ; 2.69). Among the covariates, education of the head of household, size of the household and household presence at the distribution points had a significant effect on household ITN ownership. Indeed, households with an educated head were more likely to own an ITN compared to households headed by an uneducated person (primary level: OR 2.31, 95% CI 1.32 ; 4.05, higher level OR 7.88, 95% CI 2.21 ; 28.1). Households with more than five members were less likely to own ITN compared to those with one member. Having a member present at the distribution point during the campaign was a strong predictor for a household to own a mosquito net (OR 38.49, 95% CI 25.26 ; 58.66).

**Table 1:** Logistic regression assessing the effect of household socio-economic status on ITN ownership after the net distribution campaigns

Factors	Number of households	Number of households with at least one ITN (%)	Odd Ratios (95% CI)
Total number of households	987	650 (65.9)	

Wealth quintiles			

*Lowest*	196	117 (59.7)	1.18 (0.63; 2.22)

*Second*	197	134 (68.0)	1.44 (0.77; 2.70)

*Third*	198	134 (67.7)	1.29 (0.70; 2.36)

*Fourth*	198	138 (69.7)	1.48 (0.82; 2.69)

*Highest*	198	127 (64.1)	1

Campaign wave			

*Wave1 (May 2009)*	492	309 (62.8)	1

*Wave2 (July 2009)*	495	341 (68.9)	0.93 (0.64; 1.36)

Place of residence			

*Urban*	294	202 (68.7)	1

*Rural*	693	448 (64.6)	0.89 (0.58; 1.37)

Education level of head of household			

*None*	653	411 (62.9)	1

*Primary*	168	130 (77.4)	**2.31 (1.32; 4.05)**

*Secondary*	118	77 (65.3)	1.35 (0.71; 2.57)

*Higher*	29	24 (82.8)	**7.88 (2.21; 28.10)**

*Missing*	19	11 (57.9)	

Size of the household			

*1 member*	62	38 (61.3)	

*2-4 members*	460	315 (68.5)	0.44 (0.19; 0.99)

*5-7 members*	359	226 (63.0)	0.3 (0.12; 0.71)

*8 and more members*	106	71 (67.0)	0.3 (0.11; 0.82)

Pregnant woman in the household			

*Yes*	122	83 (68.0)	0.89 (0.50; 1.58)

*No*	865	567 (65.5)	1

Under five in the household			

*Yes*	625	415 (66.4)	1.18 (0.77; 1.79)

*No*	362	235 (64.9)	1

Household present at distribution point			

*Yes*	694	601 (86.6)	**38.49 (25.26; 58.66)**

*No*	293	49 (16.7)	

Bolded: Statistically significant (*p value *< 0.05)

**Figure 2:**
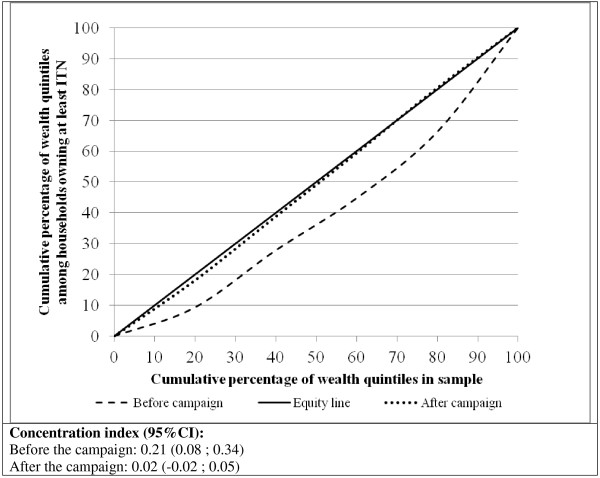
**Lorenz concentration curve assessing the equity in household ITN ownership**.

Figure 2 presents the Lorenz concentration curve, which depicts the equity in ITN ownership before (broken line) and after (dotted line) the campaign. Before the campaign the concentration curve falls far below the equity line indicating that mosquito net ownership was concentrated among households with higher socio-economic status; this is reflected in the concentration index of 0.21, 95% CI (0.08 ; 0.34). After the campaign, the concentration curve is much closer to the equity line with a concentration index of close to 0, reflecting a better equitable distribution of mosquito ITN among the different socio-economic groups.

### Household socio-economic level and mosquito net use

Table 2 presents the results of the logistic regression assessing the effect of socio-economic status on the use of ITNs. The results show that there are no statistically significant differences in mosquito net use between different socio-economic groups. Compared to the highest quintile individuals from, the lowest and second quintiles have reduced odds of using a net; however, this did not yield statistical significance (OR 0.87, 95% CI 0.67 ; 1.13 and OR 0.85, 95% CI 0.66 ; 1.10, respectively). Among all the covariates included in the model, gender is associated with use of ITN with higher odds observed among females (OR: 1.45, 95%CI 1.26 ; 1.70). Age also plays key role in ITN use with under-five child more likely to use ITN compared to older persons. The education of the head of household affects the odds of household members using ITN. Individuals from households with an educated head of household (higher education) are more likely to use ITN compared to those from households with head of household with no education (OR 1.90, 95% CI 1.23 ; 2.96). Individuals from households in which the ratio of one ITN for two members is met are more likely to use ITN (OR 2.63, 95% CI 2.06 ; 3.37).

**Table 2:** Logistic regression assessing the effect of household socio-economic status on ITNs use among individuals (de facto population) from households which own at least one ITN

Factors	Number of individuals	Number of individual using ITNs (%)	Odd Ratios (95% CI)
Totat number of individuals	3,056	1,622 (53.1)	

Wealth quintiles			

*Lowest*	521	273 (52.4)	0.87 (0.67; 1.13)

*Second*	610	307 (50.3)	0.85 (0.66; 1.10)

*Third*	640	359 (56.1)	1.1 (0.86; 1.40)

*Fourth*	653	341 (52.2)	0.91 (0.72; 1.15)

*Highest*	632	342 (54.1)	1

Gender			

*Male*	1,494	729 (48.8)	1

*Female*	1,562	893 (57.2)	**1.46 (1.26; 1.70)**

Age			

*Under 5 year*	639	397 (62.1)	1

*5-15 years*	971	471 (48.5)	**0.62 (0.50; 0.77)**

*15-25 years*	467	203 (43.5)	**0.42 (0.33; 0.55)**

*25 years and plus*	979	551 (56.3)	**0.72 (0.58; 0.89)**

Place of residence			

*Urban*	1,080	538 (49.8)	1

*Rural*	1,976	1,084 (54.9)	1.11 (0.94; 1.31)

Education level of head of household			

*None*	1,880	965 (51.3)	1

*Primary*	636	374 (58.8)	**1.39 (1.14; 1.69)**

*Secondary*	382	185 (48.4)	0.97 (0.76; 1.25)

*Higher*	115	75 (65.2)	**1.91 (1.23; 2.96)**

*Missing*	43		

Size of the household			

*1 member*	40	25 (62.5)	1

*2-4 members*	1,010	626 (62.0)	1.44 (0.74; 2.83)

*5-7 members*	1,316	670 (50.9)	1.55 (0.76; 3.14)

*8 and more members*	690	301 (43.6)	1.34 (0.66; 2.75)

Use of repellent (aerosol, coil, herbs)			

*None*	185	90 (48.6)	1

*At least one*	1,945	1086 (55.8)	1.27 (0.93; 1.75)

*At least 2*	926	446 (48.2)	0.94 (0.67; 1.32)

Ratio 1 net for 2 person met			

*No*	2,268	1077 (47.5)	1

*Yes*	788	545 (69.2)	**2.64 (2.06; 3.37)**

Bolded: Statistically significant (*p value *< 0.05)

**Figure 3:**
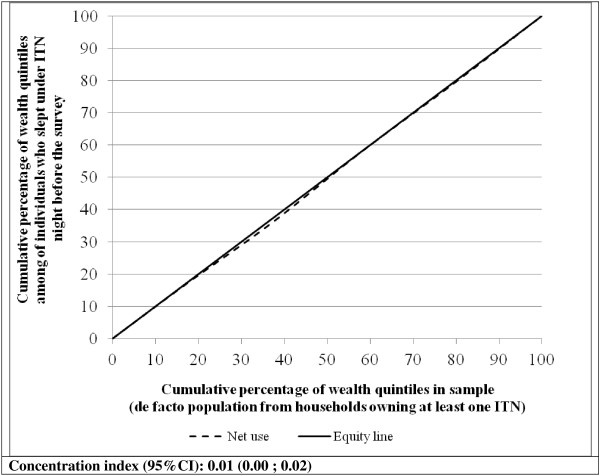
**Lorenz concentration curve assessing the equity in ITN use among individuals from households with at least one ITN**.

Figure 3 presents the Lorenz concentration curve depicting the equity in mosquito ITN use after the campaign. The curve is almost aligned to the equity line but, indicating that use of ITN among individuals from different wealth quintiles is similar. The concentration index is very close to 0 and the confidence interval includes 0 (concentration index = 0.01, 95%CI 0.00 ; 0.02).

## Discussion

Using logistic regression and equity analysis, the impact of free universal distribution of ITNs on the equity in ownership and use in Kano State was assessed. The results show that following the distribution campaigns the disparity in mosquito net ownership among households from different socio-economic groups was reduced. Comparably, mosquito net use was similar among the different socio-economic groups. No difference was observed between rural and urban populations for mosquito net ownership or use.

The two free distribution campaigns have helped to substantially increase mosquito net ownership coverage in Kano state. Before the campaigns, approximately 10% of households in Kano state owned at least one mosquito net; this low coverage is similar to national-level coverage reported in Nigeria (17%) [15]. After the campaign, coverage increased substantially to 70% in Kano state. These findings are consistent with other free mass distribution campaigns that have been carried out in sub-Saharan Africa [16-20], demonstrating that free mass distribution of ITNs can be used as an effective strategy to quickly scale-up ITN coverage in areas with low coverage. With the substantial increase in household ownership, there was concern as to whether or not the distribution campaign was also beneficial to disadvantaged subpopulations from the lowest socio-economic group. Further analyses demonstrate that coverage is equally as high among these groups.

Equity in household ITN coverage improved after the mass distribution campaigns. Prior to the campaigns, ITN ownership was greater among wealthier households as demonstrated by a positive concentration index of 0.21. After the two campaign waves, the concentration index was found to be close to zero with no statistically significant difference found between wealth quintiles in household ownership of at least one ITN. The fourth (second least poor) quintile did show higher odds for ITN ownership; however, this was not statistically significant. The findings indicate that the campaign was able to reach all households equitably and reduce the observed (before campaign) inequity in ITN ownership, suggesting a relatively fair household listing and distribution process. These results corroborate evidence from other free mass distribution campaigns that provide ITNs to the entire population at risk for malaria; all show high and equitable ITN ownership after the campaign [3,5,10,16-20].

Free mass distribution campaigns appear to be a more effective strategy to rapidly achieve high and equitable household ITN ownership coverage than other bed net distribution mechanisms, such as socially-marketed ITNs and free targeted distribution campaigns for children under five and pregnant women [10]. Strategies that involve even a nominal financial contribution from individuals are likely to benefit only those who can afford or are willing to pay for the commodity. Effective malaria control efforts using ITNs strive for high-level coverage in order to impact malaria morbidity and mortality. Therefore, free distribution seems to be an effective strategy to achieve equitable coverage at the community level that will be beneficial to everyone. High levels of ITN coverage reduce the prevalence, density and infectiousness of malaria parasites in the human population [10,21]. Other distribution strategies are less likely to achieve such high coverage, missing poorer households or those that do not have children under five or pregnant women.

ITN use was found to be relatively high after the survey, with 53.3% of individuals reporting using an ITN the night before the survey visit. However, about half of the individuals did not use ITN; this could be because of the hot weather, though absence of mosquitoes [22] could also be a factor. In northern Nigeria the annual average temperature is about 26.4 degrees Celsius; however, the average temperature was slightly higher at 27.3 degrees Celsius when the survey took place. This high temperature could cause some discomfort, especially when sleeping indoors, and some people may choose either not to use the net or to sleep outside where they are less likely to use a mosquito net. Since the hot weather is reported as a deterrent for mosquito net use in October when the temperatures are around the annual average, one should expect higher non-use in March through April when the average temperature is above 30 degrees Celsius. October corresponds to the month after the rainy season, suggesting that mosquito abundance may be low, especially if the residual mosquito breeding sites from rain water have already dried out. The nuisance of mosquitoes may have reduced substantially, resulting in reduced need to use a mosquito net. Nonetheless, the findings reveal no significant association between socio-economic status and mosquito net use after the campaign. While the lower two quintiles did show a reduced odds of ITN use, indicating greater odds of use among the wealthier quintiles, the differences were not statistically significant.

While mass distribution campaigns are able to rapidly achieve high and equitable mosquito net ownership, they are unable to provide continuous coverage for a population at risk for malaria as they only occur every few years. To provide continuous coverage, complimentary mosquito net distribution strategies need to be in place to reach people that are missed by campaigns. Such strategies could cover newborns and pregnant women between campaigns [5,21,23] as well as immigrants in areas with high population mobility. Targeted bed net distribution through antenatal care or through mass drug administration or vaccination campaigns can provide effective coverage in between campaigns to ensure that newborns and pregnant women are covered [4,6-9,16,24,25].

It is critical that increased ITN ownership and use be sustained to keep the disease burden low and potentially move towards eradication and elimination. Therefore, having complimentary bed net delivery strategies is necessary for replacing nets that are worn or damaged during the time in between mass distribution campaigns. These strategies should focus primarily on targeting poorer households, as poorer households are less likely to be able to or willing to replace worn or damaged bed nets or retreat bed nets compared to wealthier households [26]. Furthermore, bed nets could be more likely to get damaged more quickly in poorer and rural households than in wealthier or urban households due to the living conditions. Wealthier households may also be less prone to selling or trading their bed nets for immediate household needs [27]. For all of these reasons, additional ITN delivery strategies are necessary to complement mass distribution campaigns to ensure continuous and equitable coverage.

Further study to assess the survival time of the mosquito net and the factors associated with will help define a cost effective strategy for replacement in order to sustain achievements of free distribution campaigns. It will also be critical that the level of funding in malaria control be maintained or increased to sustain the free distribution strategy.

### Methodological limitations

This study presents strong evidence of the positive impact of free mosquito net distribution campaigns; however, further assessment would have been possible if baseline data were available, especially for net use. This analysis of net use was restricted to the post campaign survey data and reference to coverage before the campaign was limited to DHS 2008 data which were collected at different administrative levels. It is therefore important to conduct a pre-campaign assessment whenever possible to provide a reference indicator for the post-campaign assessment.

Fieldwork for this survey started approximately 5 months after the completion of Wave 1 of the LLIN distribution campaign and 3 months after Wave 2. It is possible that households in Wave 1 could have had more difficulty recalling the events surrounding the distribution of nets. Additionally, net retention may have been affected by the additional time between net distribution and the survey.

## Competing interests

The authors declare that they have no competing interests.

## Authors' contributions

All authors contributed to the design of the study. YY performed the analysis and wrote the first draft of the manuscript. EE, EP, SD, AK contributed to the write up and reviewed the final draft. All authors read and approved the final manuscript.
